# Usefulness of morphometric image analysis with Sirius Red to assess interstitial fibrosis after renal transplantation from uncontrolled circulatory death donors

**DOI:** 10.1038/s41598-020-63749-3

**Published:** 2020-04-23

**Authors:** Myriam Dao, Christelle Pouliquen, Alyette Duquesne, Katia Posseme, Charlotte Mussini, Antoine Durrbach, Catherine Guettier, Hélène François, Sophie Ferlicot

**Affiliations:** 1 AP-HP, Service de Néphrologie adulte, Hôpital Necker, 75015 Paris, France; 2 Inserm UMR_S 1155, Hôpital Tenon, 75020 Paris, France; 3 Service d’Anatomie pathologique, Hôpital Foch, 92150 Suresnes, France; 4 Service de Néphrologie, CHI André Grégoire, 93100 Montreuil, France; 5 AP-HP, Service d’Anatomie et de Cytologie Pathologiques, Hôpital de Bicêtre, 94270 Le Kremlin Bicêtre, France, Hôpitaux Universitaires Paris-Saclay, Le Kremlin-Bicêtre, France; 6 AP-HP, Service de Néphrologie, Hôpital de Bicêtre, 94270 Le Kremlin Bicêtre, France, Hôpitaux Universitaires Paris-Saclay, Le Kremlin-Bicêtre, France; 7 AP-HP, Unité de Néphrologie et de Transplantation rénale, Hôpital Tenon, 4 rue de la Chine, 75020 Paris, Sorbonne Université, Paris, France

**Keywords:** Diseases, Medical research

## Abstract

Early interstitial fibrosis (IF) correlates with long-term renal graft dysfunction, highlighting the need for accurate quantification of IF. However, the currently used Banff classification exhibits some limitations. The aim of our study was to precisely describe the progression of IF after renal transplantation using a new morphometric image analysis method relying of Sirius Red staining. The morphometric analysis we developed showed high inter-observer and intra-observer reproducibility, with ICC [95% IC] of respectively 0.75 [0.67–0.81] (n = 151) and 0.88 [0.72–0.95] (n = 21). We used this method to assess IF (mIF) during the first year after the kidney transplantation from 66 uncontrolled donors after circulatory death (uDCD). Both mIF and interstitial fibrosis (ci) according to the Banff classification significantly increased the first three months after transplantation. From M3 to M12, mIF significantly increased whereas Banff classification failed to highlight increase of ci. Moreover, mIF at M12 (*p* = 0.005) correlated with mean time to graft function recovery and was significantly associated with increase of creatininemia at M12 and at last follow-up. To conclude, the new morphometric image analysis method we developed, using a routine and cheap staining, may provide valuable tool to assess IF and thus to evaluate new sources of grafts.

## Introduction

Chronic kidney disease (CKD) is a burden for Public Health and concerns millions of individuals worldwide. Kidney transplantation remains the optimal treatment for CKD, offering a better survival than dialysis and being cost-effectiveness^1–3^.

In order to increase the pool of available donors in a setting of organ shortage, grafts from uncontrolled donors after circulatory death (uDCD) have been used in France since 2006. Several studies have shown that kidneys from DCD provide almost an equal function as kidneys from donation after brain death (DBD)^4–13^. Moreover, as DBD kidney transplantation, DCD kidney transplantation is associated with increased survival of patients who have end-stage renal disease (ESRD) and are on the transplant waiting list^14^. Whereas early reports of uDCD showed good renal outcome and no increase in IF/TA compared to extended criteria DBD, other reported a very early and more severe development of IF/TA^15^ than in DBD.

Whatever the donor’s status, chronic allograft dysfunction (CAD), which is the final result of different etiological and pathogenetic conditions, remains the first cause of graft loss^16–20^. CAD corresponds to the irreversible replacement of functional renal tissue by extracellular matrix (ECM) proteins, leading to the progressive impairment of renal graft function. Among the pathological lesions observed during CAD, one of the most prominent is interstitial fibrosis and tubular atrophy (IF/TA). Other histological damages include glomerulosclerosis, splitting of glomerular capillary basement membranes and vascular intimal hyperplasia^21^. Many mechanisms which are of immunological origins or not, are involved in this multifactorial and complex process^22–26^. The current concept states that many processes leading to graft fibrosis, i.e. IF/TA, occur early after the transplantation, especially within the first few months^27^. IF/TA involves about 40% of kidney grafts at 3–6 months and up to 50% at 1 year, while renal function remains stable^28,29^, which suggests that renal function is a suboptimal and late marker of kidney graft dysfunction. Up to now, the only reliable method to assess IF/TA is the histological evaluation of a graft biopsy^30,31^ which also allows to determine specific lesions and pathogenic processes affecting the graft^22,23^. It is also well-known that early IF correlates with long-term graft dysfunction^32–34^, highlighting the need for accurate quantification of IF to better identify patients requiring specific therapeutic interventions and to determine the efficacity of such interventions. IF in kidney graft is usually graded using the Banff classification^35^. However, the Banff classification exhibits some limitations. The semi-quantitative evaluation in only 4 grades (0 to 3) prevents the precise evaluation of IF evolution and may be not sensitive enough in the early stages. Furthermore, studies have shown a wide interobserver variation in the assessment of renal graft biopsies using the Banff classification^28,36–38^.

The aim of the study was to assess the initial progression of interstitial fibrosis and tubular atrophy in kidney grafts from uDCD formerly classified as “Maastricht II” non heart beating donors, using a new image analysis method based on Sirius Red staining.

In the first part of our work, we will evaluate the accuracy, the robustness and reproducibility of our computerized analysis method (mIF) to assess IF. In the second part, mIF will be applied to assess IF during the first year after kidney transplantation from uDCD and mIF score will be correlated with clinico-biological data.

## Results

### Validation and reliability tests of the image analysis method for the quantification of interstitial fibrosis (mIF)

**Figure 1:**
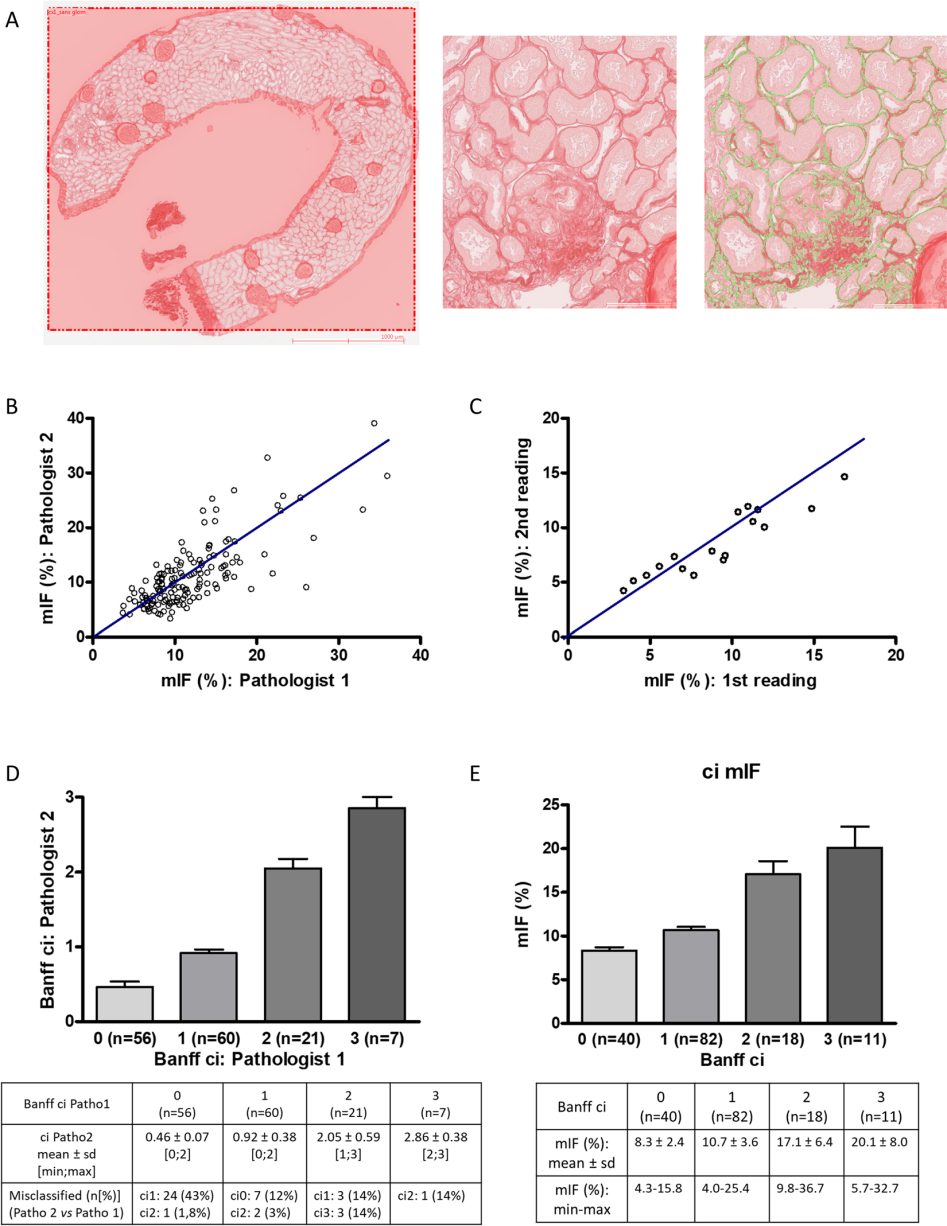
Quantification of interstitial fibrosis by image analysis: validation and reliability tests of the morphometric quantification of IF (mIF). (**A**) Morphometric analysis. Renal biopsy sections stained with Sirius red were captured by a ScanScope Aperio scanner (CS), using 20X objective. For each biopsy, the cortical section was manually selected. Glomeruli and medium-sized arteries were deleted by the operator. The red positive area was expressed as a percentage of the entire cortical kidney section using a computer-based morphometric analysis software (Calopix, Tribvn, Montrouge, France). (**B**) The value of inter-operator intraclass correlation coefficient (ICC) using mIF was 0.75 with its 95% confidence interval (95% CI) equal to [0.67–0.81] (p < 10^−3^) (n = 151). (**C**) The value of intra-observer ICC of mIF was 0.88 with 95% CI of [0.72–0.95] (p < 10^−3^) (21 consecutive graft biopsies were analyzed again 6 months later). (**D**) The value of inter-operator ICC using Banff criteria was 0.78 with 95% CI of [0.70–0.84] (p < 10^−3^) (n = 151). (**E**) mIF according to Banff ci. Pearson correlation between mIF and Banff ci was 0.62 with 95% CI of [0.51–0.71] (p < 10^−3^). Abbreviations: ci = interstitial cortical fibrosis; mIF, morphometric interstitial fibrosis; sd, standard deviation.

Among the 166 graft biopsies, 15 were rejected for image analysis because specimen adequacy was unsatisfactory (<7 glomeruli and/or no artery on the sample) according to the Banff criteria^39^ or because the paraffin block was worn out. Measurement of interstitial fibrosis by image analysis (mIF) was performed on the 151 biopsies independently by two pathologists (CP and SF) (Fig. 1A). The value of inter-operator intraclass correlation coefficient (ICC) was 0.75 with its 95% confidence interval (95% CI) equal to [0.67–0.81] (*p* = 8.4.10^-29^) (Fig. 1B). To assess the intra-observer reproducibility, 21 consecutive graft biopsies were analyzed again by one of the pathologists (CP) 6 months later. The value of ICC was 0.88 with 95% CI of [0.72–0.95] (*p* = 5.5.10^-8^) (Fig. 1C). Analysis of interstitial fibrosis (ci) according to the Banff criteria (using Masson’s trichrome staining) exhibited inter-operator ICC of 0.78 with 95% CI of [0.70–0.84] (*p* = 5.6.10^−25^) (Fig. 1D). Then, we compared morphometric quantification of interstitial fibrosis and semi-quantitative analysis performed by an expert pathologist (SF) according to Banff criteria (Fig. 1E). The mIF score significantly increased (*p* < 0.05) between the four groups defined by the Banff classification. Mean mIF (%) was respectively 8.3 ± 2.4 (ranges: 4.3–15.8) in the ci0 group (n = 40), 10.7 ± 3.6 (ranges: 4.0–25.2) in the ci1 group (n = 82), 17.1 ± 6.4 (ranges: 9.8–36.6) in the ci2 group (n = 18) and 20.1 ± 8.0 (ranges: 5.7–32.7) in the ci3 group (n = 11). The correlation between mIF and Banff ci was 0.62 with 95% CI of [0.51–0.71] (*p* < 0.001). Discrepancies between ci and mIF were observed in only 2 cases. These cases were carefully reanalyzed: interstitial edema and inflammation were the main drawback to accurately quantify ci in Massons’ trichrome staining (Supplemental Fig. 1).

### Clinical characteristics of the patients

The 66 patients who received kidney graft from uDCD included 14 females and 52 males. Mean age at the time of kidney transplantation was 44.7 ± 9.8 years old (median: 46; range: 20–59). Indications for kidney transplantation were hypertensive nephroangiosclerosis (n = 16), IgA nephropathy (n = 7), autosomal dominant polycystic kidney disease (n = 6), Alport syndrome (n = 4), diabetic nephropathy (n = 3), other glomerulopathies (n = 5), tubulo-interstitial nephritis (n = 3) and uropathy (n = 4). Nephropathy remained undetermined in 18 patients. All patients received induction therapy with anti-lymphocyte serum. They also received mycophenolate mofetil, corticosteroids and tacrolimus per local practice. Characteristics of the recipient patients are summarized in Table 1.

**Table 1:** Characteristics of donors, recipients and grafts.

Donors characteristics (n = 48)		
Age (years)*		39.7 ± 8.5
Sex: male [n(%)]/female [n(%)]		39(81%)/9(19%)
BMI (kg/m²)*		26.0 ± 4.5
Smoke history [n(%)]		28(58%)
Diabete mellitus [n(%)]		0(0%)
High blood pressure [n(%)]		0(%)
Dyslipidemia [n(%)]		3(6%)
Ischemic times	No flow time (min)*	6.9 ± 8.0
	Low flow time (min)*	138.7 ± 14.4
	Total warm ischemic time (min)*	145.9 ± 15.2
	*In situ* cold perfusion time (min)*	148.7 ± 54.1
**Recipients characteristics (n** = **66)**		
Age (years)*		44.7 ± 9.8
Sex: male [n(%)]/female [n(%)]		52(79%) / 14(21%)
ESRD cause	Nephroangiosclerosis [n(%)]	16(24%)
	Diabetes mellitus [n(%)]	3(5%)
	Glomerulopathies [n(%)]	16(24%)
	Tubulo-interstitial nephritis [n(%)]	4(6%)
	Polycystic kidney disease [n(%)]	6(9%)
	Malformative uropathies [n(%)]	4(6%)
	Unknown [n(%)]	17(26%)
Dialysis duration (months)*^,a^		32.2 ± 28.8
PRA < 10% [n(%)]		66(100%)
**Graft characteristics (n** = **66)**		
HLA mismatches (A + B + DR)*		4.5 ± 1.4
Machine perfusion [n(%)]		66(100%)
Rc lifeport at 30 min		0.21 ± 0.07
Cold ischemic time (hours)*		16.2 ± 3.7
**Clinical outcome (n** = **66)**		
Delayed graft function [n(%)]		54(82%)
Time before graft recovery (days)*^,b^		22.6 ± 9.8
Biopsy-proven acute rejection [n(%)]		8(12%)

Abbreviations: ESRD, End-stage renal disease; HLA, Human Leukocytes Antibodies; PRA, panel reactive antibodies.

*Numeric data are expressed by mean ± standard deviation.

^a^Preemptive transplantation: n = 3.

^b^Non-primary graft function: n = 2.

### Uncontrolled DCD: Baseline characteristics, transplantation and clinical outcome

The 66 patients received kidney graft from 48 uDCD (Table 1). Donors included 9 females and 39 males and were 39.7 ± 8.5 years old (median: 40.5; range: 19–53). None of them had history of arterial hypertension or diabetes mellitus. Mean total warm ischemic time (tWIT) from cardiac arrest to *in situ* preservation was 145.9 ± 15.2 minutes (median: 148.5; ranges: 115–188). Mean cold ischemia was 16.2 ± 3.7 hours (median: 17.0, ranges: 10–23.8). Delayed graft function occurred in 54/66 patients (82%). Mean time between transplantation and renal graft recovery was 22.6 ± 9.8 days (median: 20, ranges: 1–58). Non-primary graft function occurred in 2 patients. Biopsy-proven acute rejection occurred in eight patients (12%). Mean LDH level reached a pic at Day 3 post transplantation (2539 ± 1089 IU/L) which was significantly associated with time to graft recovery (time to recovery according to LDH at D3: y = 15.2 + 0.0025x, *p* = 0.016, R² = 0.1028).

### Histological features

**Figure 2:**
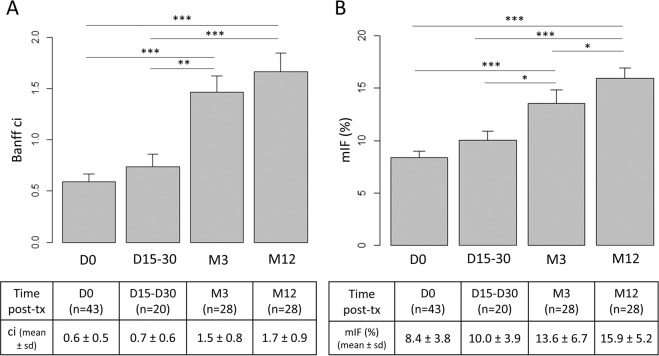
Interstitial fibrosis (IF) significantly increased after renal graft transplantation. (**A**) According to the Banff criteria, ci remained unchanged from D0 to D15/30, increased from D0 to M3 (p < 10^−3^) and remained stable between M3 and M12 (p = 0.37). (**B**) Using morphometry analysis, mIF tended to increase as early as the first month after renal transplant (p = 0.056), increase was significant from D0 to M3 (p < 10^−3^) and remained significant from M3 to M12 (p = 0.021). Abbreviations: ci = interstitial cortical fibrosis; mIF, morphometric interstitial fibrosis; sd, standard deviation; tx, renal transplantation; *p < 0,05; ***p< 10^−3^. Figure was performed using R software^58^.

Among the 48 uDCD, 43 underwent kidney biopsies (so called D0) before organ donation. Thereafter, 20 patients underwent kidney graft biopsy between 15 and 30 days (D15–30) post-transplantation for delayed graft function, 28 at 3 months (M3) and at 12 months (M12) for routine evaluation. Socio-demographic and clinical data were not significantly different between the patients who underwent biopsies at D15–30, M3 and M12. Histological features of biopsies are summarized in Table 2. Acute tubular injury significantly decreased from D0 to D15-D30 (63% ± 28% versus 47% ± 22%, p = 0.036), then to M3 (9% ± 11%, p < 10^-3^) and M12 (4% ± 6%, *p* = 0.016). Conversely, chronic lesions, especially interstitial fibrosis and tubular atrophy, significantly increased over time. According to the Banff staging system, interstitial fibrosis remained unchanged from D0 to D15/30 (0.6 ± 0.5 at D0, 0.7 ± 0.6 at D15/30), increased from D0 to M3 (0.6 ± 0.5 versus 1.5 ± 0.8, *p* < 10^−3^) and remained stable between M3 and M12 (1.7 ± 0.9 versus 1.5 ± 0.8, *p* = 0.37) (Fig. 2A).

**Table 2:** Histological features of renal biopsies at D0, D15-D30, M3 and M12.

	D0 (n = 43)	D15-D30 (n = 20)	M3 (n = 28)	M12 (n = 28)	*p* value
**Acute lesions:**					
Glomerulitis « g »	0 ± 0	0.2 ± 0.4	0.08 ± 0.3	0.07 ± 0.3	0.12
Peritubular capillaritis « ptc »	0 ± 0	0.1 ± 0.3	0.08 ± 0.3	0.04 ± 0.2	0.41
Interstitial inflammation « i »	0 ± 0	0.2 ± 0.7	0.3 ± 0.5	0.07 ± 0.3	0.01
Total inflammation « ti »	0.1 ± 0.3	0.2 ± 0.7	0.6 ± 0.8	0.8 ± 0.8	<0.001
Tubulitis « t »	0 ± 0	0.2 ± 0.7	0.2 ± 0.4	0.1 ± 0.4	0.03
Intimal arteritis « v »	0 ± 0	0 ± 0	0.04 ± 0.2	0.04 ± 0.2	0.54
Acute tubular injury (%)	63 ± 28	47 ± 22	9 ± 11	4 ± 6	<0.05
**Chronic lesions:**					
Sclerotic glomeruli	3.5 ± 5.1	2.0 ± 3.5	4.4 ± 6.7	9.0 ± 14.0	0.11
Allograft glomerulopathy « cg »	0 ± 0	0 ± 0	0 ± 0	0.04 ± 0.2	0.37
Mesangial matrix increase « mm »	0 ± 0	0 ± 0	0.1 ± 0.3	0.2 ± 0.5	0.01
Interstitial fibrosis « ci »	0.6 ± 0.5	0.7 ± 0.6	1.5 ± 0.8	1.7 ± 0.9	<0.001
Tubular atrophy « ct »	0.2 ± 0.5	0.4 ± 0.5	1.2 ± 0.8	1.5 ± 0.9	<0.001
Vascular fibrous intimal thickening « cv »	0.3 ± 0.5	0.8 ± 0.9	1.0 ± 1.1	0.7 ± 0.9	<0.01
Arteriolar hyaline thickening « ah »	0.3 ± 0.5	0.2 ± 0.4	0.4 ± 0.6	0.4 ± 0.6	0.49
**C4d (immunofluorescence):**					
Negative (0 or minimal 1)	37/37(100%)^a^	15/17(88%)^b^	24/25(96%)^b^	25/26(96%)^c^	0.22
Positive (focal 2 or diffuse 3)	0/37(0%)^a^	2/17(12%)^b^	1/25(4%)^b^	1/26(4%)^c^	0.22
**Rejection:**					
Antibody-mediated rejection (n)	NA	2^d^	1^e^	1^e^	
Borderline rejection (n)	NA	0	4	3	
T-cell-mediated rejection (n)	NA	1 ^f^	0	1 ^g^	
**Other lesions:**					

Digital data are means ± standard deviation.

^a^NA = 6.

^b^NA = 3.

^c^NA = 2.

^d^Antibody-mediated rejection included: 1 acute antibody-mediated rejection, and 1 “suspicious” for acute antibody-mediated rejection.

^e^acute antibody-mediated rejection.

^f^grade Ib.

^g^chronic T-cell mediated rejection.

### mIF significantly increased after the transplantation and correlated with clinical outcome

Image analysis IF was 8.4% ± 3.8% at D0, 10.0% ± 3.9% at D15-D30, 13.6% ± 6.7% at M3 and 15.9% ± 5.2% at M12 (Fig. 2B). Increase was significant from D0 to M3 (*p* < 10^−3^). Conversely to ci according to the Banff scoring system, increase of mIF remained significant from M3 to M12 (*p* = 0.021). Fibrosis tended to increase as early as the first month after renal transplant (*p* = 0.056). Most of the patients underwent more than one kidney biopsy. Among them, seven patients underwent early kidney graft biopsy between D7 and D30 and protocol biopsies at M3 and at M12 (Supplemental Fig. 2). In these paired cases, mIF significantly increased from first month to M3 (respectively 7.8% ± 1.8% and 11.4% ± 2.7%, p < 0.01) then from M3 to M12 (respectively 11.4% ± 2.7% and 17.7 ± 4.8%, p = 0.02). In these cases, Banff classification failed to demonstrate early increase of ci from first month to M3 (respectively 0.88 ± 0.35 and 1.3 ± 0.46, p = 0.08).

Baseline characteristics of uDCD (sex, age), tWIT and cold ischemia were not associated with mIF at any time. mIF at M12 (R² = 0.29, *p* = 0.005) but not at D0, D15–30 and M3 correlated with mean time to renal graft function recovery. In addition, mIF at M12 correlated with increase of creatininemia at M12 (R² = 0.32, *p* = 0.013) and at last follow-up (R² = 0.29, *p* = 0.005). We found the same correlation using the Banff scoring system, however, whereas there was no correlation between ci according to the Banff scoring system at M3 and creatininemia at M12 (R² = 0.15, *p* = 0.18), mIF at M3 tended to be associated with increase of creatininemia at M12 (R² = 0.31, *p* = 0.057).

## Discussion

The aim of this study was to precisely describe the evolution of IF after renal transplantation with uDCD, using a new computerized image analysis method. Indeed, IF is one of the most prominent pathological lesions of CAD and correlated with renal graft prognosis^32–34^ but the currently used Banff classification is a semi-quantitative system which prevents precise description of IF evolution and may not be sensitive enough in early stages. Slight but significant increase of IF on renal graft may be missed. Grimm *et al*. demonstrated that a precise quantification of IF by computerized image analysis provides a better surrogate marker for time to graft failure than any combination of chronic lesion scoring using the Banff schema^34^. Moreover, some studies also exhibited a wide inter-observer variation in the IF assessment according to the Banff classification^36,37^. The morphometric analysis we developed exhibited high inter-observer and intra-observer reproducibility, with ICC [95% IC] of respectively 0.79 [0.74–0.83] and 0.88 [0.72–0.95]. A variety of techniques have been used to measure renal fibrosis, each with its own advantages and disadvantages. We decided to assess IF with unpolarized Sirius red staining, firstly because Sirius red staining is commonly used in pathological laboratories since the 1980s^40^, especially to assess liver fibrosis^41–45^, secondly because to its high specificity for collagen fibers and its previously demonstrated superiority compared to polarized Sirius Red and trichrome staining^46–49^. Indeed, IF is typically considered to be an excess accumulation of fibrillary collagen. Types I and III collagen represent almost all collagens synthetized by fibroblasts and often predominate in fibrotic scars. Sirius red dye molecules intercalate into the tertiary groove in the structure of types I and III collagen and are strongly birefringent when observed under polarized light. Polarized Sirius Red analysis exhibited good results in IF assessment^34,49,50^ but it requires individual fields to be photographed in polarized light and analyzed or cost-expensive whole slide scanners capable of polarized light. Similarly, trichrome staining, that is commonly used to the visual assessment of collagen content in the interstitium, has also be used to quantify IF by image analysis, with good results^51,52^. However, studies demonstrated than unpolarized Sirius Red had not only the best correlation with estimated glomerular filtration rate (GFR)^47,48^ compared to polarized Sirius Red and trichrome staining, but also significant correlation with the decrease of GFR^33^.

In our uDCD cohort, both mIF and ci according to the Banff classification significantly increased the first three months after renal transplantation From M3 to M12, mIF significantly increased whereas Banff classification failed to highlight increase of ci. Then, mIF may be more accurate than semiquantitative Banff evaluation to identify the effects of antifibrotic therapeutic interventions. Morphometric IF tended to increase in the first month after renal transplantation but without reaching significance, due to the limited size of sample, graft biopsies being not systematically performed during the first month. However, this early and sensitive quantification of IF may be a very valuable surrogate marker to study therapeutic intervention aiming the early development if CAD in kidney transplantation. Moreover, whereas increase of creatininemia at M12 was not associated with ci according to the Banff scoring system at M3, it tended to be associated with mIF at M3, suggesting usefulness of a more sensitive quantification analysis.

In our study, mIF at M12 was significantly associated with increase of creatininemia at M12 and at last follow-up, which confirms the need for a precise assessment of IF.

IF, which corresponds to the replacement of renal functional tissue by ECM, is not only a major concern after kidney transplantation, but also during CKD in native kidneys. Indeed, renal fibrosis still represents the final target to treat CKD^53^. Surprisingly, few studies have focused on morphometric quantification of IF on native kidneys^54,55^. Hunter *et al*.^54^ demonstrated than high collagen matrix index and fibrillary collagen index, both assessed by quantitative morphometry after Sirius Red staining, predicted relapse and progression to ESRD during lupus nephritis. Similarly, Gibyeli Genek *et al*.^55^ performed a quantitative evaluation of IF with Sirius Red in IgA nephritis and highlighted that such evaluation might serve as an effective novel method to determine the prognosis in IgA nephritis.

To conclude, the new morphometric image analysis method we developed, using a routine and cheap staining, may provide valuable tool to assess IF during chronic allograft dysfunction and thus to evaluate new sources of grafts. The use of this method to describe CKD on native kidney should also be evaluated.

## Methods

### Patients

After institutional review board, we retrospectively included all the 66 patients who received a kidney graft from uDCD between 2007 and 2012 in Bicêtre hospital. Kidney donation followed the 2008 Declaration of Istanbul principles and the French Agence Nationale de la Biomédecine regulation. Research was approved by the committee of the Centre de Ressources Biologiques (CRB, Paris Sud University). Informed written consent was given by all the patients for the scientific use of the graft biopsies in the CRB, Paris Sud University. All procedures and the use of tissues were performed in accordance with the Declaration of Helsinki principles. Clinical reports and biological data were collected from the associated clinical database.

### Histological review of kidney graft biopsies

A total of 166 kidney graft biopsies was performed in the 66 patients. The biopsies were processed for routine light microscopy, as we previously described^56^. Biopsy samples were fixed in formalin, acetic acid and alcohol (AFA), paraffin-embedded and sliced 2.5 µm thick. Slides were stained with HES (hematoxylin, eosin and saffron), Masson’s trichrome (3 sections), periodic acid Schiff, Jones methenamine silver and Sirius red. Third Masson’s trichrome and Sirius red staining were consecutive. Kidney graft biopsies were reviewed independently by 2 pathologists (CP and SF) for histological features according to Banff recommendations^35^ in a blind manner from clinical data. Immunostaining for C4d was performed using a rabbit monoclonal A24-T anti-human C4d antibody (DB Biotech, Kosice, Slovak Republic; dilution 1/100) and a Leica BOND-MAX™ autostainer (Leica Biosystems Newcastle Ltd, UK). Epitope retrieval was achieved using the ready-to-use Bond Epitope Retrieval Solution 1 (Leica Biosystems Newcastle Ltd, UK) after 30 min heating.

### Quantification of interstitial fibrosis by image analysis

Renal biopsy sections sliced 2.5 µm sliced and stained with Sirius red were digitalized by a ScanScope Aperio scanner (CS), using 20X objective. For each biopsy, the cortical section, defined as the part inside the renal capsule and outside the medulla, was manually selected on digital slides. Glomeruli and medium-sized arteries were deleted by the operator.

Renal-cortex fibrosis was quantified using a computer-based morphometric analysis software (Calopix, Tribvn Healthcare, Châtillon France) as previously published^56,57^. Briefly, the operator manually selects internal Sirius Red negative areas and positive areas and next runs the software that show the final selection of the Red area. The internal negative control step allows the comparison of slides that are sequentially stained in various batches of Sirius Red since staining usually may vary. Finally, the result was expressed as a percentage of the red positive area on the total cortical surface. Morphometric analyses were performed twice for each biopsy in an independant manner by two pathologists (CP and SF) who had no knowledge of the clinical data.

### Statistical analyzes

Descriptive statistical methods (means, medians, standard deviations and ranges) were used to assess the distributions of variables. Wilcoxon rank sum and *t* tests for continuous variables, Fisher’s exact and chi-squared tests for categorical variables were performed. The intraclass correlation coefficient (ICC) with its 95% CI was used to study the reproducibility of morphometric IF (mIF) measures. Correlations between quantitative variables were assessed with Pearson product-moment correlation coefficient. For all analyses, a *p* value <0.05 was regarded as significant. Analyses were performed using R software (version 3.2.0)^58,59^, InStat 3 software (GraphPad Software, San Diego, CA) and Prism 4 (GraphPad Software).

## Supplementary Information

Supplementary Information.

## Data Availability

The datasets generated during and/or analyzed during the current study are available from the corresponding author on reasonable request.
