# Supplementary material for: Usefulness of morphometric image analysis with Sirius Red to assess interstitial fibrosis after renal transplantation from uncontrolled circulatory death donors
Source: Sci Rep. 2020 Apr 23;10:6894. doi: 10.1038/s41598-020-63749-3 (PMC7181605; doi:10.1038/s41598-020-63749-3)
Supplement: Supplementary file 1 — Supplementary Information. [file 41598_2020_63749_MOESM1_ESM.pdf]

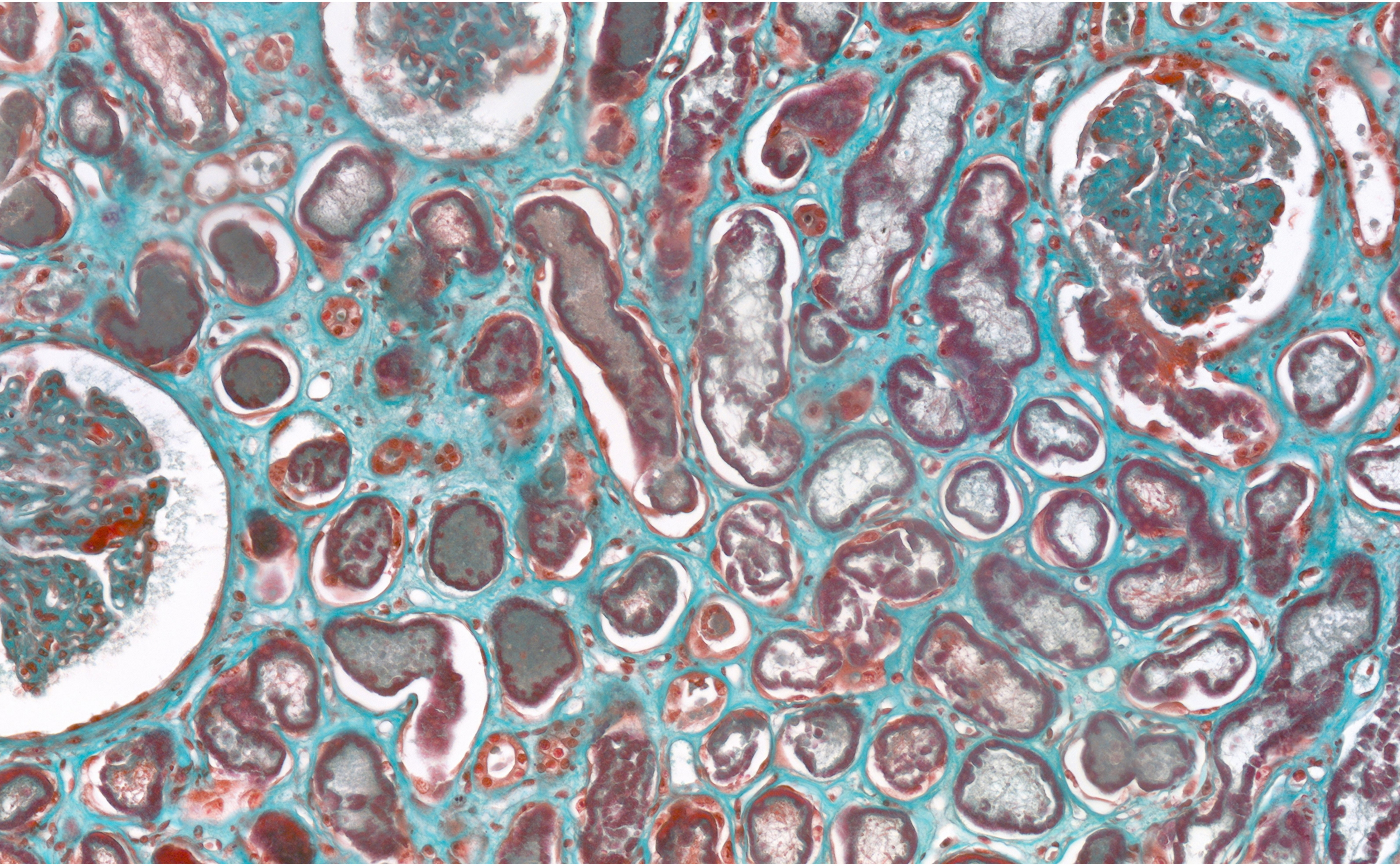

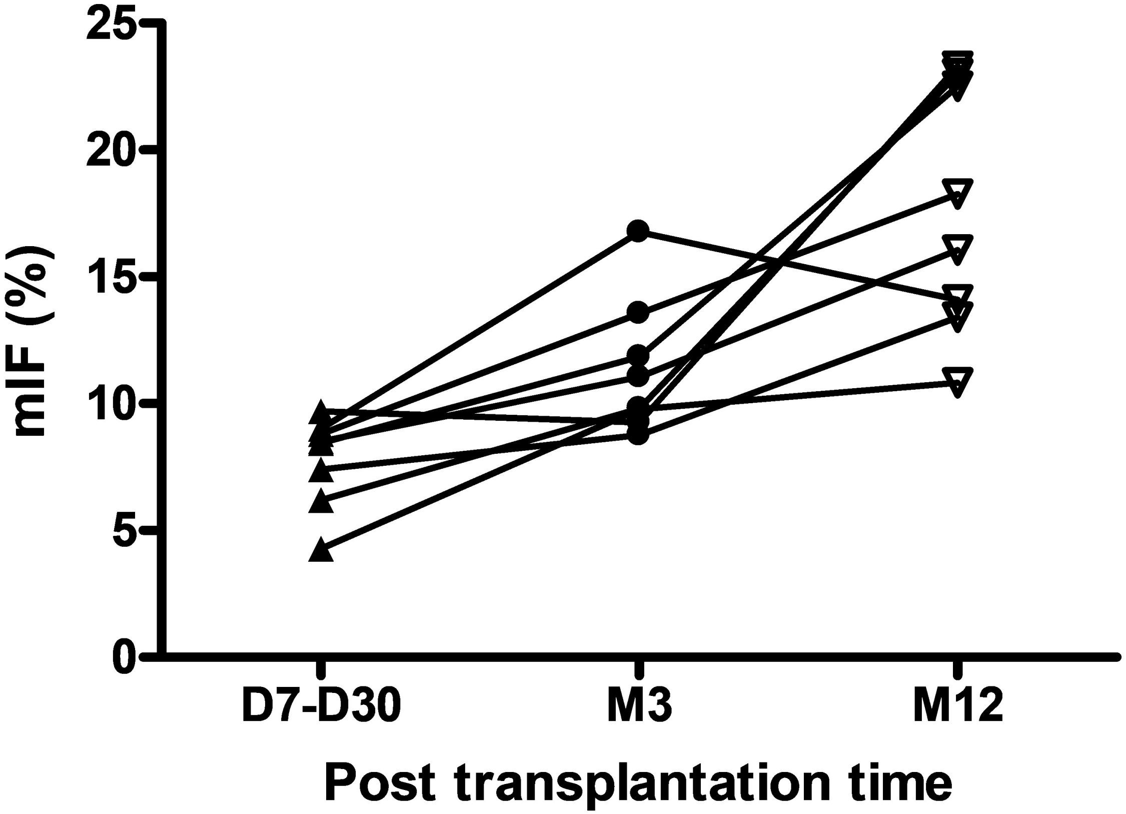

	D7-D30	M3	M12
mIF (%): mean ± sd	7.8 ± 1.8	11.4 ± 2.7	17.7 ± 4.8
ci: mean ± sd	0.88 ± 0.35	1.3 ± 0.46	2.0 ± 0.93

Usefulness of morphometric image analysis with Sirius Red to assess interstitial fibrosis after renal transplantation from uncontrolled circulatory death donors

Myriam Dao[+,1,2], Christelle Pouliquen[+,3], Alyette Duquesne[4], Katia Posseme[5], Charlotte Mussini[5], Antoine Durrbach[6], Catherine Guettier[5], Hélène François[§,2,7,*] and Sophie Ferlicot[§,5]

[+]Myriam Dao and Christelle Pouliquen contributed equally to the work.

[§]Hélène François and Sophie Ferlicot contributed equally to the work.

[1] AP-HP ; Service de Néphrologie adulte, Hôpital Necker, 75015 Paris, France

[2] Inserm UMR_S 1155, Hôpital Tenon, 75020 Paris, France

[3] Service d'Anatomie pathologique, Hôpital Foch, 92150 Suresnes, France

[4] Service de Néphrologie, CHI André Grégoire, 93100 Montreuil, France

[5] AP-HP ; Service d'Anatomie et de cytologie pathologiques, Hôpital de Bicêtre, 94270 Le Kremlin Bicêtre, France ; Hôpitaux Universitaires Paris-Saclay

[6] AP-HP ; Service de Néphrologie, Hôpital de Bicêtre, 94270 Le Kremlin Bicêtre, France ; Hôpitaux Universitaires Paris-Saclay

[7] AP-HP ; Unité de Néphrologie et de Transplantation rénale, Hôpital Tenon, 4 rue de la Chine, 75020 Paris ; Sorbonne Université.

* Corresponding author: helene.francois@aphp.fr

Supplemental Figure 1 - Interstitial edema and acute tubular necrosis prevented accurate quantification of interstitial fibrosis in Massons' trichrome staining. This illustrative case was performed in a 52-y-old woman. She received a kidney graft from uDCD and underwent kidney graft biopsy at day 9 after the transplantation to investigate delayed graft function. Graft biopsy exhibited thrombotic microangiopathy in the glomeruli, diffuse interstitial edema and acute tubular necrosis. Interstitial edema disjoined the tubules. Also, ci was rated 2 with IF/TA grade 2 in Massons' trichrome staining. Conversely, morphometric quantification allowed to refine biopsy analysis, with mIF at 11.3%.

Supplemental Figure 2 – Morphometric image analysis with Sirius Red allowed to detect early increase of interstitial fibrosis renal transplantation. Seven patients underwent early kidney graft biopsy between D7 and D30 and protocol biopsies at M3 and at M12: mIF significantly increased from first month to M3 ($p < 0.01$) then from M3 to M12 ($p = 0.02$). Conversely, Banff classification failed to demonstrate early increase of ci from first month to M3. Abbreviations: ci = interstitial cortical fibrosis; mIF, morphometric interstitial fibrosis; sd, standard deviation.
